# Status quo of genetic improvement in local goats: a review

**DOI:** 10.5194/aab-65-207-2022

**Published:** 2022-05-20

**Authors:** Glafiro Torres-Hernández, Jorge Alonso Maldonado-Jáquez, Lorenzo Danilo Granados-Rivera, Homero Salinas-González, Gabriela Castillo-Hernández

**Affiliations:** 1 Colegio de Postgraduados-Campus Montecillo, 56230 Montecillo, Estado de México, México; 2 Instituto Nacional de Investigaciones Forestales, Agrícolas y Pecuarias, Centro de Investigación Regional Norte Centro, Campo Experimental La Laguna, 27440 Matamoros, Coahuila, México; 3 Instituto Nacional de Investigaciones Forestales, Agrícolas y Pecuarias, Centro de Investigación Regional Noreste, Campo Experimental General Terán, 67400 General Terán, Nuevo León, México; 4 Cooperative Extension and Research, Lincoln University, 65101 Jefferson City, MO, USA; 5 Facultad de Estudios Superiores Cuautitlán, Universidad Nacional Autónoma de México, 54714 Cuautitlán Izcalli, Estado de México, México

## Abstract

This review aims to summarize and synthesize the fragmented information available on the genetic improvement of local goats (criollo, indigenous, native) on the American and other continents, where populations with these goats have an important role in food security and the economy of rural communities, as well as in conservation of biodiversity and productivity improvement. Topics such as the current state of goat production globally, conservation programs, resistance to parasites and diseases, use of phenotypical characteristics and genomic information, and molecular markers for genetic improvement are addressed. The main challenges, opportunities, and limitations described in recent literature concerning local goats in the immediate future are discussed.

## 1 Introduction

Goats have been a food source for humans for many years, since their expansion throughout the world (Sevane et al., 2018). Worldwide, there are around 1094 million goats; most of them (94 %) are in countries of Asia and Africa, followed by the Americas (3.4 %) and Europe (1.4 %) (FAOSTAT, 2019). In China, there are around 58 domestic local breeds, distributed in different environments (Du et al., 2011). India has 23 well-recognized goat breeds (Mandal et al., 2014). In Africa, most of the goats (63 % of the population) are non-improved native breeds (Visser and van Marle-Köster, 2017), and in the Americas, there are around 28 recognized local breeds (Boettcher et al., 2014). In addition, the majority of these populations are utilized for self-consumption of products such as milk, meat, and skin or fibers, and they are found in herds that belong to low-income producers (Dubeuf and Boyazoglu, 2009).

On the other hand, the available information about genetic improvement (GI) of local goats (Montaldo et al., 2010), also known as criollo, indigenous, or native, in the Americas and other regions of the world is limited; however, the available results indicate that local breeds have a high potential for improvement (Argyriadou et al., 2020). In addition, the variation between and within these breeds is highly valuable, since great genetic variability is required for the populations to adapt and face the environmental changes that they are subjected to (Ojo et al., 2015). In time, GI has been limited to the “commercial” breeds, although their low participation in the animal registry and improvement plans has resulted in slow progress and an accelerated loss of genetic variability (Visser and van Marle-Köster, 2017).

In this sense, the incipient generation of information about populations of local goats is due, in principle, to the fact that there is the notion that they have a lower productive and economic performance, which decreases interest among producers and places a specific breed or genotype at risk of extinction (Biscarini et al., 2015). Therefore, the greatest challenge of GI in local breeds is the adaptation of selection schemes and classic improvement in small populations and production systems characterized by poor organization from producers (Gandini et al., 2017). In addition, it is necessary to include the ideology of small-scale producers in the objectives and selection criteria. For example, Gunia et al. (2010) mention that the conformation and growth of males are among of the most desirable traits for producers in the Caribbean (77 % of the interview respondents), followed by maternal behavior, reproduction, and milk production. They conclude that GI programs must consider the use of local populations as maternal lines.

### 1.1 Use of exotic germplasm and its impact on local populations

In countries like Iraq, Syria, Morocco, and Tunisia, efforts have been directed to design GI programs, which have consisted of the distribution of pure breed bucks (Saanen, Nubian, Toggenburg, Alpine, etc.). So, this has not had the foreseen success due to the low adaptation of bucks to the environment. In addition, there is limited participation of producers in the design of GI plans (Iñiguez-Rojas et al., 2013). Consequently, farmers are not motivated to invest in these programs, resulting in intermittent implementation and low success (Argyriadou et al., 2020). This situation is similar in Latin American countries such as Mexico (Montaldo et al., 2010; Alejandre-Ortiz et al., 2016), Ecuador (Gómez-Carpio et al., 2016), Peru (Gómez-Urviola et al., 2016), and Bolivia (Stemmer and Valle Zárate, 2016).

The goats that migrated to the “new world” (the West) have European, African, and Central Asian origins, from which the local goats found in the Americas are derived and represent a reservoir of genetic diversity to be used in breeding programs, selection, and conservation (Paim et al., 2019). Genetic improvement has been done in Brazil by introducing goat populations from Portugal and Spain and more recently from Asia, Africa, and Europe (Braga-Lôbo et al., 2010). This caused these goats to go through intense adaptation processes to overcome severe climate conditions in this country, particularly in the northeast of Brazil, thus originating a particular goat type that is clearly separate from the other local breeds of the rest of the American continent, with a marked influence from Cape Verde goats (Sevane et al., 2018).

Deza (2007) proposed a genetic improvement plan in Argentina for local goats in “agroecologically restrictive” environments, stemming from the characteristics attributed to the local goat. Among these characteristics, the following stand out: animals that are polymorphic, polychromic, rustic, and adapted to extreme environments, which have not been sufficiently described and therefore have still-unknown productive potential, with high productive variability between and within the herds. For this reason, most of the efforts to conduct GI in these populations continue to be done traditionally (Ahmed et al., 2020).

### 1.2 Community-based breeding programs

In countries such as Bangladesh, community-based breeding programs (CBBPs) have been established, whereby, contrary to what was mentioned before, producers participate with the common interest of conserving and improving their genetic resources in low-income production systems (Bhuiyan et al., 2017). Liberia also incorporated a pilot CBBP in three countries with the participation of goat farmers (Karnuah et al., 2018a). In Ethiopia, the implementation of this GI plan increased producers' income by 20 % by improving the size of the litter. In addition, meat consumption at the farm level increased, so a CBBP is considered a feasible plan with measurable genetic gains in performance characteristics and the impact on the standard of living of producers (Haile et al., 2020).

In Turkey, positive effects were observed beyond improving the animals, since the living standard of producers and some market situations improved; however, a critical success factor for these programs is the monitoring and continuous training of the producer (Saatci et al., 2017). Likewise, Mueller (2017) evaluates the characteristics of some CBBP cases for the production of dairy goats in Mexico, Cashmere goats in Iran, Mohair goats in Argentina, and dairy goats in Kenya. The study concludes that planning and participation by local institutions are of utmost importance for support in the organization, financing, and technical support of the participating producers. Since one of the main problems faced by this strategy is sustainability in time, GI efforts for local goats must be conducted from an integral perspective (Wurzinger et al., 2021).

For example, in Malawi and Uganda, the use of CBBPs increased growth performance and survival of offspring; however, to achieve adoption of this model, it is recommended to establish producer cooperatives and reform regional and supportive policies (Kaumbata et al., 2021). Also, it must inevitably correlate the existing genotypes with the environment without underestimating its potential for improvement and emphasizing the need to make more significant conservation efforts, such as what has been proposed in Kenya (Rewe et al., 2002). Given the variability of CBBP success in Ethiopia, valuable recommendations have been generated that could improve the impact of these programs. The actions to be implemented are reproductive improvements such as artificial insemination and synchronization of oestrus, which will allow knowing the pedigree of the animals, removal of the effect of the birth season, and acceleration of the breeding process with the best identified individuals (Weldemariam and Mezgebe, 2021).

### 1.3 Conservation programs

Knowledge of the genetic structure of organisms, genetic populations, and phylogenetic studies is essential to provide opinions about effective conservation and reintroduction plans, mainly when the target population is at risk of extinction (Ariyaranthe et al., 2016). For the specific case of local goats, these have high significance due to their unique adaptation characteristics, with a particular relevance because of their low maintenance, which makes them a vital ally to face and combat the effects of climate change (Monau et al., 2020). In this sense, some characterization efforts in Sri Lanka, Slovenia, and Greece have found that local goat populations have a distinct genetic identity and are heterogeneous. The contemporary structure of the population is highly influenced by anthropogenic actions, making it necessary to establish immediate activities for their conservation (Ariyaranthe et al., 2016; Michailidou et al., 2019; Pogorevc et al., 2021). However, the lack of emphasis on production based on technology and appropriate characterization of breeds remains a limiting factor for the sustainable use of these goats (Monau et al., 2020).

Liu et al. (2019) conducted a study to define conservation priorities of 26 local breeds in China to establish protection programs according to the importance within and between breeds given by genetic diversity, and they found that the Inner Mongolia Cashmere, Jining Gray, and Liaoning Cashmere goats were the ones that contributed most to heterozygosity and total diversity. However, for the case of Daiyun and Shannan Blanca goats, their conservation should be prioritized based on the population's effective size.

Due to the low productive indices of local goats in Brazil, “exotic” breeds were introduced, which, even though they are more productive, do not have adaptation characteristics (resistance to diseases and parasites) that local goats have. Despite this fact, exotic breeds have substituted local breed (Canindé, Gurguéia, Moxotó, Marota, and Repartida), placing the latter in danger of extinction. To avoid the loss of this genetic resource, the Program for Conservation of Animal Genetic Resources was created through “conservation nuclei” in habitats where the goats are subject to natural selection (in situ) and also from storage of semen and embryos (ex situ) (Mariante et al., 1999).

In Cuba, an exploration of 48 herds that were thought to be “local” was performed, finding that only one of them was indeed local. Therefore, a research–action process was implemented for the conservation of this zoogenetic resource, with the active participation of farmers facing an adverse environment with social complexities (Chacón-Marcheco et al., 2016). Similarly, this effort has been carried out in countries like Mexico (Salinas-González et al., 2011, 2013), where the conservation process resulted in a fundamental scenario for the development of several studies, which have contributed valuable information related to genetic characteristics. With this learning, peasant systems acquired new development opportunities, but they conserved the socioeconomic rationalities for breeding, whose capacity to “change and conserve at the same time” was called “socioecological resilience” (Chacón-Marcheco et al., 2016). Within this context of innovation for conservation, there were community livestock fairs held as a tool to revitalize the participative management of zoogenetic resources. This strategy eases the flow of specimens through purchase–sale practices or solidary practices of exchange and loan.

Another conservation program for local goats that has entailed good planning, organization, and monitoring has taken place in the French Antilles (Naves et al., 2016) and to a lesser degree in Ecuador (Gómez-Carpio et al., 2016). In Mexico, without official programs, the conservation of local goats in some regions has been found to be a necessity by the producers themselves, who have confirmed the advantages of local genotypes compared to the improved breeds that have been introduced. Some examples are the Tarahumara goat in Sierra de Chihuahua (Alejandre-Ortiz et al., 2016), the White Celtiberian goat in Zacatecas (Reveles-Torres et al., 2008; Sánchez-Gutiérrez et al., 2021), the local goats in Comarca Lagunera (Escareño-Sánchez et al., 2011; Maldonado-Jáquez et al., 2018), the Black goats in Querétaro (Andrade-Montemayor, 2017), and the Pastoreña goat from the Mixteca regions in Puebla and Oaxaca (García-Bonilla et al., 2018; Villarreal-Arellano et al., 2020).

Likewise, in France, the interest in the conservation of biodiversity of domestic animals, including goats, has been “rekindled”, since a fast decrease or even disappearance of certain breeds has been observed. Therefore, the choice of “returning” to grazing as an option to conserve these local zoogenetic resources is being studied, since historically extensive livestock production has been linked to local aspects, traditional breeds, and therefore direct conservation (Bertaglia et al., 2005).

## 2 Resistance to parasites and diseases

### 2.1 Resistance to gastrointestinal nematodes

The most significant efforts for research on this topic have been made with local goats from the French Antilles (de la Chevrotière et al., 2011; Bambou et al., 2013; Mandonnet et al., 2014). However, Brazil maintains an improvement program between breeds that also includes resistance to nematodes (Costa et al., 2000).

Infections due to gastrointestinal nematodes (GINs) in small ruminants represent the main limitation of production in grazing systems in the tropics because they cause economic losses when decreasing the production (Miller and Horohov, 2006; Preston et al., 2014). In that regard, anthelmintic products to control GINs had success for many years; however, resistance to these products has spread throughout the world.

Concerning this, Costa et al. (2000) studied the variability between and within goat breeds by measuring the number of eggs per gram of feces (EPG), packed cell volume (PCV), and hemoglobin (HB) in local kids of the breeds Canindé and Bhuj, as well as in Nubia, all exposed to *Haemonchus contortus*. The results indicated a negative correlation between egg counts and blood values and suggest differences between breeds in PCV and HB; this, in turn, is related to resistance to infection by *H. contortus* or the effects.

Another alternative to counteract the problem of parasitosis from gastrointestinal nematodes is dietary supplementation. In this regard, Torres-Acosta et al. (2006) studied the effect of dietary supplementation in local kids that graze on native vegetation during the humid season in Yucatán, Mexico, on resilience to infections by GINs. The results showed that dietary supplementation improved the kids' resilience to GIN infections, which, in addition, is an economically viable technology.

Creole goats from the Guadeloupe islands in the Caribbean are one of the most studied breeds regarding resistance to infections by GINs. This genetic resistance has been studied since 1995 because they are very susceptible to these infections. In this regard, Mandonnet et al. (2001) studied the genetic variation for resistance to infection from nematodes to introduce this variable into genetic improvement plans. The researchers found a heritability value ( $h^{2}$ ) for EPG of 0.37±0.06 at weaning and between 0.14±0.05 and 0.33±0.06 at 4 and 10 months during fattening. The $h^{2}$ for PCV varied from 0.10 to 0.33 with maternal and direct $h^{2}$ values at weaning for EPG of 0.26 and 0.20, respectively; genetic correlations between PCV and body weight decreased from 0.47 to 0.10 between weaning and 10 months of age, when the kids are infected by *Haemonchus contortus* and *Trichostrongylus colubriformis*, showing that the GI for resistance to GINs is viable.

In their review, Mandonnet et al. (2014) addressed the appropriateness and viability of performing the selection of local goats based on their resistance to GINs and developed three strategies to face this problem: (a) reducing the host's contact with the infectious larvae through a reduced load in grazing, (b) extending the efficiency of molecules from synthetic anthelmintic molecules by implementing directed selective treatments or the use of phytotherapeutic drugs, and (c) increasing the ability of the host to tolerate the negative effects of the worms (resilience) and eventually responding to the parasites (resistance) from genetic selection.

The problem of infections from GINs has been studied at the molecular genetic level. In this regard, a large number of loci for quantitative characteristics have been detected (QTLs) associated with resistance to GINs in small ruminants in more than 20 chromosomal regions (Dominik, 2005; Bishop and Morris, 2007). The first scan of the genome for resistance to GINs in goats was carried out in local goats from Guadeloupe (de la Chevrotière et al., 2011) and identified 13 QTLs for resistance, resilience, and immunity criteria. Later, after performing a quality control analysis of SNPs (single-nucleotide polymorphisms), they identified 46 643 markers for studies by GWASs (genome-wide association studies) (Silva et al., 2018).

Likewise, Aguilar-Caballero et al. (2008), in their review, highlighted the existence of resilience and resistance as natural defense mechanisms of local goats against GINs. In addition, they discussed the situation of gastrointestinal parasitosis in goats, the control measures applied, their current situation, and the alternative methods for GINs control-tested in goats that can be transferred to the field (Aguilar-Caballero et al., 2011).

### 2.2 Resistance to diseases

Disease resistance is a topic in which the component of genetic resistance has been studied as part of control mechanisms, not only because of its impact on productivity but also because of the zoonotic and economic implications that it represents (Buhari et al., 2020; Palomares-Reséndiz et al., 2021). In this sense, this genetic resistance is considered an alternative for the control of diseases and the use of antimicrobials; it includes both immune and non-immune mechanisms and is defined as the inherent capacity of an animal not previously exposed to pathogens. It has been found that natural resistance is inherited and passed from parents to offspring (Adams and Templeton, 1998). This paper will consider some studies that have evaluated the genetic resistance of local goats to various diseases.

Brucellosis is a zoonotic disease of public importance in countries such as Mexico, Nigeria, and Algeria, which is caused by the bacterium *Brucella melitensis*. Regarding this disease, transmission patterns and dynamics have been studied, as have some induced and natural resistance mechanisms. To date, it has been found that genetic resistance to intracellular pathogens is linked to a genomic region of the SLC11A1 gene, which is considered a candidate for resistance to this disease (Sahraoui et al., 2020; Palomares-Reséndiz et al., 2021).

On the other hand, hydrocarditis or aqueous heart (“cowdriosis” or “heartwater”) is an acute infectious disease that is non-contagious and fatal. It is produced by rickettsiae in ruminants, caused by *Ehrlichia ruminantium*, and transmitted by Amblyomma ticks. So, it occurs in nearly all the countries of Africa and nearby islands, as well as the Caribbean. The disease can cause high mortality in susceptible domestic ruminants (up to 90 %); goats and sheep are more vulnerable than cattle and European breeds more than African ones (Teklu et al., 2017; Dinkisa, 2018). In this regard, Bensaid et al. (1993) suspected resistance to this disease in Creole goats from Guadeloupe, and therefore Bambou et al. (2010) performed a study to confirm this resistance and susceptibility in local goats to a standardized sub-lethal infection of *E. ruminantum*, inducing 70 % mortality. They measured the intensity of the disease using clinical reaction indices (incubation period, fever intensity, nervous signs, and death), and differences were found between the mortality rate after infection. This was the first study wherein genetic variability of this disease was found, and there is an attempt to link this variability physically with areas of the genome. Likewise, Matheron et al. (1987) concluded that resistance seems to be under genetic control since a herd studied for 10 years and exposed to the disease reached a resistance rate of 78 %.

Even more, the goat arthritis encephalitis (GAE) is a chronic disease eased by a retrovirus of the Lentivirus genus. There are no vaccines or effective treatments. Therefore, the hypothesis has been suggested that through genetic selection, there could be a feasible alternative for the control of this disease, as reported by Schultz et al. (2020), who estimated heritability values of 0.077 (with ranges of 0.026 to 0.128) for resistance to GAE in Alpine and Saanen goats.

Kim et al. (2019) found candidate genes for resistance to Salmonella (LBP and BPI) and heart disease (TTN and ITGB6) in Korean, Iranian, and Moroccan goats, which is of maximum importance to direct future efforts to conserve species biodiversity and contribute to genomic improvement programs. For their part, Meydan et al. (2017), identified resistant genotypes in variants of the gene that encodes the prion protein (K ${}_{222}$ ), observing a decrease in “tremors” caused by Scrapie's disease. This resistance has been observed in local goats from Italy (Migliore et al., 2015).

## 3 Phenotype of local goats as selection criteria in genetic improvement programs

Phenotypical characterization is the first phase to document the qualitative and quantitative characteristics of local goats. In this regard, a large variety of colors, types of beards, horns, ears, and hair, among others, has been reported (Table 1). Most of the variations observed in phenotypical characterization studies are due to environmental factors, topography, and vegetation, which give rise to different ecotypes (Monau et al., 2020). In addition, it fosters differences between and within breeds (Table 2). For example, the withers height of Bolivian local goats (51–58 cm) is lower when compared to that of Sabanero local goats from Colombia (75–80 cm). The great difference in body length between local goats from Arque, Bolivia (47 cm), and Amatepec, Mexico (104 cm), is also worth noting, representing a difference higher than 100 %. Even among the same local goats, there are differences for these same characteristics (Stemmer and Valle-Zárate, 2016).

**Table 1:** Phaneroptic characteristics in local goats from various countries of the world.

Genotype	Location	PH (%)	TE (%)	CC (%)	PB (%)	MC (%)
Cuban ${}^{a}$	Sierra Maestra, Cuba	100	Erect (71 %) Semi-erect (29 %)	1 color (28 %) 2 colors (44 %) 3 colors (29 %)	100	Black (57 %) Pink (43 %)
Criollo ${}^{b}$	Córdoba, Argentina	100	Hanging	White	–	–
Local ${}^{c}$	Coahuila, Mexico	65.2	–	Black (15 %) White (20 %)	97.8	Black (61 %) Pink (35 %)
Criollo ${}^{d}$	Peru	100	Short straight	Varied	–	With pigments
Criollo ${}^{e}$	Bolivia	92.4	Erect and semi-erect	Black and white	–	–
Chinchorrera ${}^{f}$	Oaxaca, México	–	Semi-erect	Black, white, brown	–	–
Nondescript ${}^{g}$	Kashmir, India	10	Long and droopy (78 %) Short (22 %)	Black (24 %) Brown (27 %) White (15 %)	36	–
West African Dwarf ${}^{h}$	Liberia	70	Erect and semi-pendulous	Black and white (63 %)	–	–
Indigenous ${}^{i}$	Sidama, Ethiopia	100	Semi-erect (71 %) Dropped (24 %)	White (50 %) Brown (10 %)	49	–
Local ${}^{j}$	Morocco	70	Erect (84 %) Dropped (14 %)	Black (38 %) Red (30 %) White (14 %)	75	–
Indigenous ${}^{k}$	Ghana	76	Horizontal (48 %) Erect (40 %)	Brown (27 %) Black (27 %) Multiple (35 %)	18	–

PH: presence of horns; TE: type of ears; CC: coat color; PB: presence of beard; MC: mucosa color; ${}^{a}$ Chacón et al. (2011), ${}^{b}$ Deza et al. (2007), ${}^{c}$ Moyao-Ariza et al. (2022), ${}^{d}$ Gómez-Urviola et al. (2016), ${}^{e}$ Stemmer and Valle-Zárate (2016); ${}^{f}$ Ortiz-Morales et al. (2021), ${}^{g}$ Ali Rather et al. (2020); ${}^{h}$ Karnuah et al. (2018b), ${}^{i}$ Hankamo et al. (2020), ${}^{j}$ El Moutchou et al. (2017), ${}^{k}$ Hagan et al. (2012).

In this sense, the external characteristics of local goats are an essential selection criterion that must be considered, since they are the first impression of the animal. It is a critical point to ensure the dissemination of the genetic material of those valuable animals and to guarantee appropriate genetic progress (Labatut et al., 2013). In this regard, producers from Patagonia, Argentina, and Oromia (Ethiopia) use several morphometric and phaneroptic criteria to select their replacements, highlighting the size, animal conformation, type of hair, and coat color (Lanari et al., 2005; Bedada et al., 2019).

**Table 2:** Morphometric characteristics (mean ± SD, cm) of local goats in diverse regions of the world.

Genotype	Location	HL	WH	BL	TP	EL
Cuban Criollo ${}^{a}$	Cuba	17.7	60.9	65.5	76.8	12.6
Local ${}^{b}$	Amatepec, Mexico	20.8	66.2	104	–	18.3
Native of Formosa ${}^{c}$	Formosa, Argentina	20.6	62.0	70.4	82.4	–
White Celtiberian ${}^{d}$	Zacatecas, Mexico	22.9	63.6	78.5	81.4	16.2
Criollo ${}^{e}$	Ecuador	22	59	75	78	14
Criollo ${}^{f}$	Bolivia	–	51-58	47	65-71	13
Pernakan ${}^{g}$	Indonesia	–	74.4	75.3	80.9	–
Kacang ${}^{g}$	Indonesia	–	62.3	60.8	69.3	–
Bligon ${}^{g}$	Indonesia	–	73.5	62.6	72.9	–
Local ${}^{h}$	Algeria	18.8	70.9	73.8	63.9	16.6
Migratory ${}^{i}$	Junagadh, India	22.0	80.6	75.4	84.5	–
Sahelian ${}^{j}$	Katsina, Nigeria	–	66.4	75.2	73.3	–
Indigenous ${}^{k}$	South Africa	–	64.8	73.8	80.7	–
Shami ${}^{l}$	Syria	–	80.0	76.1	90.8	20.4
Hamra ${}^{m}$	Morocco	28.0	64.8	61.5	40.9	–

HL: head length; WH: withers height; BL: body length; TP: thoracic perimeter; EL: ear length; ${}^{a}$ Chacón et al. (2011), ${}^{b}$ Dorantes-Coronado et al. (2015), ${}^{c}$ Prieto et al. (2006), ${}^{d}$ Sánchez-Gutiérrez et al. (2018), ${}^{e}$ Gómez-Carpio et al. (2016), ${}^{f}$ Stemmer and Valle-Zárate (2016), ${}^{g}$ Alawiansyah et al. (2020), ${}^{h}$ Benyoub et al. (2018), ${}^{i}$ Patbandha et al. (2018), ${}^{j}$ Rotimi et al. (2020), ${}^{k}$ Tyasi et al. (2020); ${}^{l}$ Hassen et al. (2016), ${}^{m}$ Hilal et al. (2016).

Valencia-Posadas et al. (2017) evaluated the phenotypical relations between conformation and milk production traits and reported that goats with better body conformation were not the best to produce milk, which can be directly related to the productive aptitude of the genotype in question and should therefore be considered at the time of implementing GI schemes. Because of this, a new methodology has been developed that combines the use of phenotypical distribution models to predict the behavior of the livestock, and it has been concluded that it might be a highly valuable tool to evaluate and conserve the breeds that adjust better to specific environments (Lozano-Jaramillo et al., 2019).

Undoubtedly, data collection in productive phenotypical characteristics from producers, particularly under field conditions, represents devoting time, effort, and concentration, which in many cases is not enough to have a reliable database for their later use in GI programs. In this sense, phenomics, understood as the application of technologies that allow the collection of phenotypes easily, economically, and in large volumes, represents a great opportunity to achieve considerable improvements (Mrode et al., 2020), since, with the widespread growth of communication technologies, GI programs for local populations could be revolutionized in developing countries. Some examples include the use of sensors in females to detect patterns of reproductive behavior, the prediction of body weight in animals for meat production using a measuring tape and measurements of the thoracic perimeter, and the use of mobile devices that help to evaluate milk production and weight gain, among others (Mrode et al., 2020).

## 4 Use of genomic and molecular tools in local goat breeding

High-density genomic tools have been used in the last decade to study and characterize livestock's genetic diversity and population structure. However, many individual phenotypes and genotypes are required to obtain accurate genetic values, which in the case of local populations with low numbers of individuals can only expect low genetic gains (Biscarini et al., 2015). A large variety of techniques has been developed to help solve this problem in order to evaluate variability and diversity at the DNA level (Table 3). Nowadays, techniques are available, such as random amplified polymorphism DNA (RAPD), amplified fragment length polymorphism (AFLP), restriction fragment length polymorphism (RFLP), simple sequence repeat (SSR), and short tandem repeats (STRs), among others. According to the literature consulted, the most frequently used techniques to estimate the genetic diversity between populations of sheep, goats, bovines, buffaloes, camels, and horses are RAPD (Al-Barzinji and Hamad, 2017) and microsatellite markers or STRs (Arangurén-Méndez et al., 2005).

On the other hand, genome-wide association studies (GWASs) help to explore the relationship between genetic and phenotypic variation in terms of genomic markers, identifying variants that can be part, or not, of known genes. Stemming from this, molecular research has opened new possibilities for the genetic characterization, conservation, and utilization of the only genetic resource that local goats have (Visser and van Marle-Köster, 2017; Wittenburg et al., 2020). However, the use of these tools has not been implemented for goats on a broad scale. In that regard, in Greece, studies of this type in native goats are limited. Only microsatellite markers or SNPs have been used to characterize genetic distances between breeds based on polymorphisms of blood proteins, finding significant genetic variability and potential for improvement in productive and aptitude traits (Argyriadou et al., 2020). In Italy, studies with SNPs have been carried out to characterize the genetic diversity of native dairy goats such as Argentata dell'Etna, Derivata di Siria, Girgentana, and Messinese goats (Di Gerlando et al., 2020). Likewise, Ceccobelly et al. (2020) mention that molecular tools are of great help to discriminate breeds adequately at a low cost. In addition, they must be considered to trace goat products and distinguish their origin.

In India, Jayashree et al. (2019) conducted a study to identify the diversity of Karnakata goats through 23 microsatellite markers and found a heterogeneous local population, which is highly valuable because of its adaptation characteristics. Likewise, in Sri Lanka, many localized native goats cover most of the country's goat population and are at high risk of extinction from intensive crossing with exotic breeds. This situation positions them as an invaluable genetic resource from their high environmental adaptation and resistance to diseases. Therefore, SNPs have been used to identify regions of the genome that are correlated with the productivity, resistance, and fertility of goats (Ariyaranthe et al., 2017).

Based on the preliminary information, the use of these genomic and molecular tools has become of utmost importance for the improvement and conservation of native breeds in general, since they allow combining pedigree, phenotypes, and genotypes in a simple evaluation without the need for post-analysis processes (Lourenco et al., 2020).

In their review, Mrode et al. (2018) indicated that CBGI is a reasonable frame of reference; innovative genomic selection will be required in fragile growth systems wherein adaptation is an important characteristic, and it will be essential to identify regions of the genome related to aspects of adaptability with the aim of maximizing the diversity of animals. In addition, the same authors (Mrode et al., 2018) point out that an adequate cost–benefit analysis ought to be part of any strategy to implement genomic selection in these production systems.

An example of the use of these techniques is reported by Sevane et al. (2018), since they generated a perspective of the diversity of goats in the Americas using genomic tools, by using information from Iberian, African, and local breeds. For this case, they obtained important signatures of Iberian breeds, particularly Cuban Criollo, but with a significant contribution from African breeds that have given rise to many local breeds. With this information, there are advances in revealing the evolutionary history of local goats and defining conservation priorities to maintain the diversity that they have inherited.

**Table 3:** Genomic–molecular studies carried out with local goats in various regions of the world.

Country	Genotype	Type of marker	Characteristic evaluated
Tunisia ${}^{a}$	Local	Microsatellite	Polymorphism of Casein Alpha-S1 gene (CSN1S1)
Mozambique ${}^{b}$	Indigenous	Microsatellite	Genetic diversity
Zimbabwe ${}^{b}$	Indigenous	SNPs	Resistance to gastrointestinal parasites
Nigeria ${}^{c}$	Red Sokoto Kano Brown	Microsatellite	Genetic diversity
Algeria ${}^{d}$	Native	Microsatellite	Polymorphism of SLC11A1 gene for brucellosis resistance
India ${}^{e}$	Assam local	Microsatellite	Genetic diversity
Indonesia ${}^{f}$	Local	Microsatellite	Genetic diversity
India ${}^{g}$	Nandidurga and Bidri	Microsatellite	Genetic diversity
Italy ${}^{h}$	Garfagnina	Genomic	Genetic diversity
Brazil ${}^{i}$	Moxotó, Canindé, Serrana Azul, Repartida, Garúna, Marota	Microsatellite	Genetic diversity
Mexico ${}^{j}$	Celtiberian White	RAPD	Genetic diversity
Russia ${}^{k}$	Local	SNPs	Genetic diversity
America ${}^{*,l}$	Creole	Microsatellite	Genetic variability
Uganda ${}^{m}$	Mubende, Kigezi, Karamonog, Sebei	SNPs	Breed homozygosity

${}^{a}$ Jemmali et al. (2012), ${}^{b}$ Monau et al. (2020), ${}^{c}$ Ojo et al. (2015), ${}^{d}$ Sahraoui et al. (2020), ${}^{e}$ Zaman et al. (2014); ${}^{f}$ Zein et al. (2012), ${}^{g}$ Tantia et al. (2018), ${}^{h}$ Dadousis et al. (2021), ${}^{i}$ Menezes et al. (2020), ${}^{j}$ Reveles-Torres et al. (2008), ${}^{k}$ Deniskova et al. (2021), ${}^{l}$ Ginja et al. (2017), ${}^{m}$ Onzima et al. (2018). ${}^{*}$ Sample of 910 animals from 10 countries.

## 5 Challenges, limitations, and opportunities of genetic improvement in local goats

### 5.1 Challenges

During the last century, erosion of goat genetic resources has been observed as a result of a massive replacement of “low-productivity” local breeds by other “highly productive” specialized breeds (Di Gerlando et al., 2020). However, the statement that specialized breeds are more productive is not entirely correct, since there is evidence suggesting that crossbreeding with pure breeds does not necessarily improve the productive aptitude of local genotypes under the management conditions in which these genotypes are traditionally developed. This way, the overall result is lower production than expected from the interaction of an unfavorable genotype with the environment (Lozano-Jaramillo et al., 2019). Evidence of this fact was given by Gaddour et al. (2010), who found that crosses of Damasco with Alpine and Murciano–Granadina goats improved milk production and kid growth, but negative effects in reproductive traits were also detected.

Due to the above, producers of local goats face the challenge of maintaining or increasing the size of their populations and thus avoiding extinction. Therefore, strategies should be sought that help apply all available tools to improve the productivity and profitability of these farms, including a wide range of GI technologies like genomic profiles, targeted genetic improvement schemes, and different reproductive, marketing, and traceability tools (Biscarini et al., 2015; Michailidou et al., 2019) that could help small-scale producers to survive. On the other hand, a critical challenge faced by producers is the availability of technical and scientific information, since, in general, information on new technological advances is available mostly among the scientific community; for producers, this information is massively disseminated because in some parts of the world, access to this type of information is highly restricted (Argyriadou et al., 2020) or the cost to access it is extremely high, which seriously affects decision-making in public policies and at the farm level. The abovementioned issue is not exclusive to rural areas, but this lack of information is also present in urban and metropolitan areas; therefore, the challenge is to generate strategies whereby new information on technologies and/or findings in any aspect of the production chain reaches producers and decision makers so that they can be applied. In addition, another of the great challenges observed is to seek strategies that help sustainability and mitigate the effects of climate change to guarantee the supply of food of animal origin to a growing population (Wattiaux, 2019).

### 5.2 Limitations

A severe limitation in GI programs for these populations is the lack of continuity between research and practical implementation, which is aggravated by the deficiency of resources to monitor these programs due in principle to the fact that government research and development programs have only considered productivity, without considering aspects of population adaptation. In this sense, some research objectives are altered based on scientific trends, resulting in intermittent studies without future efforts (Argyriadou et al., 2020). In that regard, Djemali (2000) points out that in countries such as Tunisia, those who have invested for decades in GI programs for local goats to be successful have found that governments hardly support animal registry and selection costs for prolonged periods. In addition, there is a scant connection between state entities that implement GI programs and researchers who are the ones with the “know-how” about what should be used. Furthermore, involvement from producers is not observed, leading to constant changes in the objective's selection, thus limiting the genetic progress. An example of the failure of these GI projects, wherein the producer is not considered, happened with goat producers in Morocco and Senegal (Kosgey et al., 2006). In other words, there is a will to improve the production schemes but a lack of real organization of all the actors involved. Concerning this, Giovannini (2011) suggested that the greatest limitations for the transference of GI plans are inadequate policies, precarious economic development, problems inherent to production systems, precarious institutional development, and precarious and unsustainable financing.

### 5.3 Opportunities

One of the great opportunities presented by local goats is exceptional genetic diversity and variability, which is essential to adapt production systems to future challenges and represents a critical point to face climate change and its effects (Paim et al., 2019).

Advances have been found for reproductive traits. Since the estimators of heritability for these traits are low, huge amounts of data are required for the GI process to be effective, which is complicated in local populations (Atoui et al., 2018). However, researchers can develop strategies to improve reproduction in local goat genotypes, given the advancement in genomic and molecular tools (Dagong et al., 2020). This could allow identifying genomic–genetic variants with relative ease, which would make it possible to apply scanning techniques of the genome, association studies in the genome, and genomic selection to increase prolificacy (Gomes de Lima et al., 2020). In addition, high genetic variability and a low level of inbreeding in native breeds have been found, indicating that there are no negative signs for the sustainable use of goats in the future (Karsli et al., 2020).

A wide range of possibilities has been explored for GI in populations of local goats. In that regard, Atoui et al. (2020) pointed out that some management practices seem to have an impact on estimating genetic components for growth in local kids; that is, the selection at critical ages that defines changes in the growth components seems to be more important when using frequent weighing. As pointed out by Torres-Hernández et al. (2020), this should be considered within GI programs for an accurate estimation of milk production through lactation. Although all the strategies contribute to knowledge, the main limitations for improving local goats in many parts of the world are fundamental issues such as diseases and parasites, poor nutrition (quality and quantity), genetic potential without quantifying, inadequate infrastructure and access to markets, minimal institutional and services support, and poor access and underutilization of knowledge, information, and technologies (Nwogwugwu et al., 2018).

Finally, countries such as Sudan and Italy have clearly defined the production objectives for their native genotypes and believe that the use of genomic–genetic technologies should be made widespread for the characterization of genotypes or breeds. It is thought that these would save considerable time and resources (El Hag et al., 2020) although emphasizing that special attention must be paid during the selection process to not altering the adaptation of goats to local conditions, specifically those characteristics that are of interest for conservation (Gandini et al., 2017). Therefore, an urgent need has been generated to consider different measures to address the limitations identified so far in terms of the GI of local goats to exploit the potential genetic diversity and benefit of these animals (Nwogwugwu et al., 2018).

## 6 Conclusions

Significant advances have been observed in recent years in the conservation and genetic improvement of local goats globally, primarily due to the last-generation molecular tools. However, it is still perceived that efforts are isolated and intermittent. They are still not enough to conserve and recover these populations, despite them being an invaluable reservoir of genetic material resistant to diseases and parasites that is adapted to produce and reproduce under extreme environmental conditions.

These characteristics make local goats one of the few viable and sustainable options of low economic, nutritional, and environmental requirements. They can help to recover both food self-sufficiency, with products of high biological value, and income in developing countries with extreme poverty facing the adverse effects of climate change.

Some other genetic improvement and conservation strategies for these populations should focus on the characterization of the productive system in an integral manner and where phenotypic and genotypic characterization should be considered part of the evaluation of the various mechanisms of adaptation to the environment that these goats have developed since this information can contribute to the development of programs for better comprehensive genetic improvement schemes.

Because of this, it is urgent to redouble efforts in research, support, and accompaniment in all the links of the productive chain that seek practical solutions to the significant problems presented by the production of one of the noblest animal species that has been vital for the development of humanity.

## Data Availability

The data are available upon reasonable request from the corresponding author.
